# SNP diversity of *Enterococcus faecalis *and *Enterococcus faecium *in a South East Queensland waterway, Australia, and associated antibiotic resistance gene profiles

**DOI:** 10.1186/1471-2180-11-201

**Published:** 2011-09-12

**Authors:** Irani Rathnayake, Megan Hargreaves, Flavia Huygens

**Affiliations:** 1 Cell and Molecular Biosciences, Faculty of Science and Technology, Queensland University of Technology, 2 George Street, Brisbane, 4001, Australia

## Abstract

**Background:**

*Enterococcus faecalis *and *Enterococcus faecium *are associated with faecal pollution of water, linked to swimmer-associated gastroenteritis and demonstrate a wide range of antibiotic resistance. The Coomera River is a main water source for the Pimpama-Coomera watershed and is located in South East Queensland, Australia, which is used intensively for agriculture and recreational purposes. This study investigated the diversity of *E. faecalis *and *E. faecium *using Single Nucleotide Polymorphisms (SNPs) and associated antibiotic resistance profiles.

**Results:**

Total enterococcal counts (cfu/ml) for three/six sampling sites were above the United States Environmental Protection Agency (USEPA) recommended level during rainfall periods and fall into categories B and C of the Australian National Health and Medical Research Council (NHMRC) guidelines (with a 1-10% gastrointestinal illness risk). *E. faecalis *and *E. faecium *isolates were grouped into 29 and 23 SNP profiles (validated by MLST analysis) respectively. This study showed the high diversity of *E. faecalis *and *E. faecium *over a period of two years and both human-related and human-specific SNP profiles were identified. 81.8% of *E. faecalis *and 70.21% of *E. faecium *SNP profiles were associated with genotypic and phenotypic antibiotic resistance. Gentamicin resistance was higher in *E. faecalis *(47% resistant) and harboured the *aac*(6')-*aph*(2') gene. Ciprofloxacin resistance was more common in *E. faecium *(12.7% resistant) and *gyrA *gene mutations were detected in these isolates. Tetracycline resistance was less common in both species while *tet*(L) and *tet*(M) genes were more prevalent. Ampicillin resistance was only found in *E. faecium *isolates with mutations in the *pbp5 *gene. Vancomycin resistance was not detected in any of the isolates. We found that antibiotic resistance profiles further sub-divided the SNP profiles of both *E. faecalis *and *E. faecium*.

**Conclusions:**

The distribution of *E. faecalis *and *E. faecium *genotypes is highly diverse in the Coomera River. The SNP genotyping method is rapid and robust and can be applied to study the diversity of *E. faecalis *and *E. faecium *in waterways. It can also be used to test for human-related and human-specific enterococci in water. The resolving power can be increased by including antibiotic-resistant profiles which can be used as a possible source tracking tool. This warrants further investigation.

## Background

Poor microbiological quality of water results from contamination by microorganisms of human or animal origin and leads to the risk of gastro-enteritis in humans [1,2]. The assurance of the microbiological quality of environmental water used as a source for recreational water is a global issue [3]. Total coliforms, faecal coliforms, *Escherichia coli *and enterococci are commonly used microbial indicators of water quality [4]. However, several studies of both recreational and drinking water samples suggested that enterococci are more relevant indicators of faecal contamination than faecal coliforms and *E. coli *[5,6]. Previous epidemiological studies demonstrated a correlation between the concentration of enterococci in surface waters and an increase in swimmer-associated gastroenteritis [5-8]. The United States Environmental Protection Agency (USEPA) advises various limits as guidelines for recreational water quality. For freshwater, the present single-sample advisory limit is 61 cfu/100 ml for enterococci. The 5-day geometric mean should not exceed 33 cfu/100 ml for enterococci [9]. According to the Australian National Health and Medical Research Council (NHMRC) guidelines, there are four microbial assessment categories, A-D, based on enterococcal counts per ml (A ≤ 40, B 41-200, C201-500 and D > 501) together with associated health risks [10].

Enterococci are members of the natural intestinal flora of animals and humans and are released into the environment directly or via sewage outlets [11]. Certain members of the genus, particularly *E. faecalis *and *E. faecium*, are becoming increasingly important as opportunistic pathogens [7,12,13]. Most important and a contributing factor to the pathogenesis of enterococci is their resistance to a wide range of antibiotics [14]. Enterococci have been found to be increasingly resistant to multiple anti-microbial drugs in last few years [15-17]. Enterococci show either intrinsic resistance where resistance genes are located on the chromosome, or they possess acquired resistance determinants which are located on plasmids or transposons [18]. Examples of the intrinsic antibiotic resistance include resistance to beta-lactams, cephalosporins, sulfonamides, and low levels of clindamycin and aminoglycosides [18,19]. Resistance to chloramphenicol, erythromycin, high levels of clindamycin and aminoglycosides, tetracycline, high levels of beta-lactams, fluoroquinolones, and glycopeptides such as vancomycin are examples of acquired resistance [19].

The distribution of infectious enterococcal strains into the environment via water could increase the prevalence of these strains in the human population. Environmental water quality studies may benefit from focusing on a subset of *Enterococcus *spp. that are consistently associated with sources of faecal pollution such as domestic sewage, rather than testing for the entire genus. *E. faecalis *and *E. faecium *are potentially good focal species for such studies, as they have been consistently identified as the dominant *Enterococcus *spp. in human faeces [20-22] and sewage [23]. The characterisation of *E. faecalis *and *E. faecium *is important in studying their population structures, particularly in environmental samples. Different methods have been developed for the characterisation of enterococci [24-28]. However, there is a need to develop and apply new robust, rapid and cost effective techniques which are likely to yield more definitive results for the routine monitoring of *E. faecalis *and *E. faecium*. This was addressed in our previous study where we developed a single-nucleotide polymorphisms (SNP) based genotyping method to study the population structure of *E. faecalis *and *E. faecium *[29]. A set of eight high-D SNPs was derived from the *E. faecalis *and *E. faecium *Multilocus Sequence Typing (MLST) database, using the 'Minimum SNPs' software program, which provided a high Simpson's index of diversity (D), calculated with respect to the MLST database. An allele-specific real-time PCR (AS Kinetic PCR) method was developed to interrogate these high-D SNPs [29]. SNP interrogation is an efficient means of classifying *E. faecalis *and *E. faecium *into groups that are concordant with the population structure of these organisms [29]. In this study we have applied this rapid SNP genotyping method to determine the diversity of enterococci in the Coomera River, South East Queensland, Australia over a period of two years and also investigated the antibiotic resistance determinants associated with *E. faecalis *and *E. faecium *SNP genotypes.

## Methods

### Study site

The Pimpama-Coomera watershed is located in South East Queensland, Australia and is used intensively for agriculture and recreational purposes and has a strong anthropogenic impact. The main water source is the Coomera River, which flows for 90 km from its headwaters in the Lamington National Park. The upper reaches of the river passes through mainly rural areas comprising crops and cattle grazing. In the middle to lower reaches, land uses include farming and cropping. In the 1970s and 1980s the river was widened 20 km upstream from the mouth as a consequence of sand and gravel extraction operations. The lower reaches of the Coomera River passes through highly developed areas including canal estates such as Santa Barbara, Hope Island, Sanctuary Cove and the Coomera Mooring Marina. Most of the sewage system collection is gravity fed and follows natural catchment drainage lines until the wastewater is treated at the central treatment plant. After treatment, the water is released into the Gold Coast Seaway located south of the Coomera River estuary. Despite the existence of such an effective treatment system, high numbers of coliforms were observed over a long period of time in the estuary.

### Sampling

**Figure 1:**
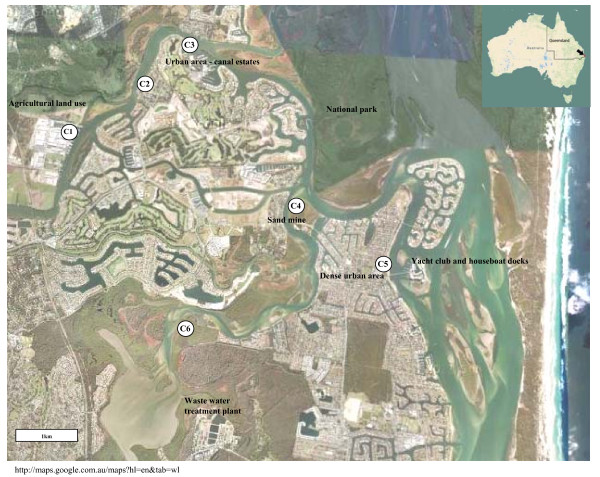
**Water sampling sites along the Coomera River, South-East Queensland, Australia**. C1 - Coomera marina, cattle/kangaroo feeding, house-boat mooring site; C2 - Santa Barbara, well used park, BBQ, toilets and fishing, private houses about 100 m away; C3 - Sanctuary Cove, canal estate, modern houses and apartments, modern infrastructure, commercial/light industrial area; C4 - Jabiru Island, busy through road, disused sand mine, no houses, small park with toilets; C5 - Paradise Point, public swimming area, mouth of river, much water traffic; C6 - Coombabah; established suburban area, bush island opposite.

Environmental water samples were collected during four seasonal trips at the same time each day from six designated sites of the Coomera River, from May 2008 to July 2009. Hot-spots selected for sampling included: Coomera Marina (C1), Santa Barbara (C2), Sanctuary Cove (C3), Jabiru Island (C4), Paradise Point (C5) and Coombabah (C6). These sites were suggested by the Gold Coast City Council as being problematic sites with a history of high numbers of faecal coliforms. The positions of these sampling sites are shown in Figure 1. The exact location and characteristics of sampling sites are summarised in Table 1.

**Table 1:** Sampling site locations and characteristics

Code	Site name (GIS^b ^map reference)	Site characteristics
C1	Coomera marina (-27.861672, 153.339089)	Cattle/kangaroo feeding, house-boat mooring site
C2	Santa Barbara (-27.855165, 153.350612)	Well used park, BBQ, toilets and fishing, private houses about 100 m away
C3	Sanctuary Cove (-27.851617, 153.362140)	Canal estate, modern houses and apartments, modern infrastructure, commercial/light industrial area
C4	Jabiru Island (-27.879057, 153.380685)	Busy through road, disused sand mine, no houses, small park with toilets
C5	Paradise Point (-27.886359, 153.396596)	Public swimming area, mouth of river, much water traffic
C6	Coombabah, Estuary (-27.896607, 153.366845)	Established suburban area, bush island opposite

^b ^Global information system

Water samples were collected in sterile bottles according to the sampling procedures described in the USEPA microbiology methods manual [9]. The sampling depth for surface water samples were 6-12 inches below the water surface. Samples were transported in a cooler on ice packs to the laboratory where they were prepared for analyses immediately upon arrival and were tested within 6 h of collection for the presence of enterococci.

### Isolation and identification of enterococci

The environmental water samples were mixed thoroughly, and undiluted samples or a 1:10 dilution of water samples were filtered through 0.45 μm membrane filters (MilliporeCorporation, Bedford, MA, USA), placed onto membrane-Enterococcus Indoxyl β-D-Glucoside Agar (mEI) (Becton-Dickinson, Sparks, MD, USA) according to the USEPA specifications [30]. Triplicate samples were collected from each site and each sample was treated separately. The addition of Indoxyl-β-D-Glucoside, Nalidixic acid, 0.1 N NaOH, and Triphenyltetrazolium Chloride to mEI agar (Difco) allowed for a single 24 h incubation period at 41°C [31]. *E. faecium *ATCC 27270, *E. faecalis *ATCC 19433 and *E. coli *ATCC 25922 were used as positive and negative controls respectively to validate the mEI agar. Colonies producing a blue halo were typically observed for enterococci and counted, the result expressed as cfu/ml for each water sample.

### Statistical analysis

The Mann-Whitney U-test at 5% significance level was performed to determine whether there was a significant increase of total enterococcal counts (cfu/ml) at each location after rainfall events.

### Identification of *E. faecium *and *E. faecalis*

Typical colonies on the membranes were identified to the genus and species level by Gram-stain, catalase test, the ability to tolerate 6.5% NaCl and biochemical tests [32]. The isolates identified as *E. faecium *and *E. faecalis *were used in this study and species identification was confirmed by performing real-time PCR to detect the *ddl_E. faecalis _*and *ddl_E. feacium _*genes. The primers used were: 5'CAAACTGTTGGCATTCCACAA3' and 5'TGGATTTCCTTTCCAGTCACTTC3' (*E. faecalis *forward and reverse primers respectively); and 5'GAAGAGCTGCTGCAAAATGCTTTAGC3' and 5'GCGCGCTTCAATTCCTTGT3' (*E. faecium *forward and reverse primers respectively) [29].

### Antibiotic susceptibility testing

Antibiotic resistance phenotypes were determined by the disc diffusion method according to the Clinical and Laboratory Standards Institute (CLSI) recommendations [33]. Saline suspensions of isolated colonies selected from an 18-24 hour Brain Heart Infusion agar (Oxoid, Australia) plates were prepared and suspension turbidity was adjusted to an equivalent of a 0.5 Mc Farland standard and inoculated onto Mueller Hinton agar (Oxoid, Australia) using sterile cotton swabs. Antibiotic discs for ampicillin (AMP, 10 μg), ciprofloxacin (CIP, 5 μg), gentamicin (GEN, 10 μg), tetracycline (TET, 30 μg), and vancomycin (VAN, 30 μg), were placed onto the surface of each inoculated plate. The diameters of antibiotic inhibition zones were measured and recorded as susceptible (S), intermediate resistant (IR) or resistant (R) according to CLSI M02-A10. *E. faecalis *ATCC 29212 and *Staphylococcus aureus *ATCC 25923 were used for quality control.

### DNA Extraction

Enterococcal strains were sub-cultured into Brain Heart Infusion broth (Oxoid, Australia) and incubated at 37°C overnight. A 400 μl aliquot of an overnight culture was used for DNA extraction. The Corbett X-tractor Gene automated DNA extraction system was used to extract DNA from all cultured isolates (Corbett Robotics, Australia) using the Core protocol No.141404 version 02. The automated DNA extraction system allows for the simultaneous extraction of DNA from 96 isolates. The quality and quantity of the DNA was high, yielding 98 ug/ml DNA on average and with a mean 260:280 absorbance ratio of 1.85.

### SNP profiling of *E. faecium *and *E. faecalis *by Allele-specific Real-Time PCR

A method for a highly-discriminatory SNP genotyping method for *E. faecium *and *E. faecalis*, has been developed by our group [29]. In total, 55 *E. faecalis *and 53 *E. faecium *isolates were genotyped by the SNP method using Allele-specific real-time PCR (RotorGene 6000, Corbett Robotics). Each reaction contained 2 μl of DNA which was added to 8 μl of reaction master mix containing 5 μl of 2 × SYBRGreen^® ^PCR Mastermix (Invitrogen, Australia) and 0.125 μl of reverse and forward primers (20 μM stock, final concentration 0.5 μM) [29]. Cycling conditions were as follows: 50°C for 2 min, 95°C for 10 minutes, followed by 40 cycles of 95°C for 15 seconds, 60°C for 60 seconds, and a melting stage of 60°C-90°C. Each isolate was tested in duplicate and No Template Controls (NTCs) were used for each primer set as well. An isolate specific SNP profile for all *E. faecium *and *E. faecalis *was generated consisting of the polymorphism present at each of the SNPs. A complete description of the relationship between the SNP profiles of each isolate and MLST-defined population structure was determined for both *E. faecalis *and *E. faecium*, using the MLST database and the "working backwards" mode of the Minimum SNPs program.

### SNP validation by sequencing of MLST housekeeping genes

*E. faecalis *and *E. faecium *isolates representing each possible SNP were used to validate the polymorphism present at each position. Sequencing was performed to confirm the SNP profiles using MLST sequencing primers listed at http://efaecalis.mlst.net/misc/info.asp and http://efaecium.mlst.net/misc/info.asp. PCR products were prepared for sequencing using the high pure PCR product purification kit (Roche, Indianapolis, USA) according to manufacturer's instructions. Between 18 -30 ng DNA template was mixed with the relevant sequencing primer at a final concentration of 9.6 pmol in a 12 μl reaction containing the Big Dye terminator mix (Australian Genome Research Facility - AGRF). Sequencing reactions were performed using a protocol of 96°C for 1 min, 96°C for 10 s, 50°C for 5 s and 60°C for 4 min on the AB3730XL platform. Sequencing data were analyzed using Chromas (version 1.43, Technelysium, Tewantin, Australia) and Vector NTI (version 11, Invitrogen, Australia) software programs.

### Real-Time PCR for the detection of antibiotic resistance

#### Primer design

Real-Time PCR primers for genes encoding vancomycin (*vanA, vanB)*, tetracycline (*tet*(L), *tet*(M), *tet*(S)), ciprofloxacin (*gyrA*), ampicillin (*pbp *5) and gentamicin (*aac*(6')-*aph*(2')) resistance were designed using the Primer Express 2.0 primer design software program (Applied BioSystems) (Table 2). Primers were synthesised by Sigma-Aldrich, Castle Hill, New South Wales, Australia.

**Table 2:** Oligonucleotide primers for Real-Time PCR detection of genes encoding for resistance to vancomycin (*vanA, vanB, vanC1, vanC2)*, tetracycline (*tet*(L), *tet*(M), *tet*(S)), ciprofloxacin (*gyrA)*, ampicillin (*pbp*5) and gentamicin (*aac*(6')-*aph*(2'))

Target gene	Primer name	Primer sequence (5' to3')	Positive control
*van A*	vanAF^a^	TGTGCGGTATTGGGAAACAG	ATCC 51559
	vanAR^b^	GATTCCGTACTGCAGCCTGATT	
*van B*	vanBF	TCTGCTTGTCATGAAAGAAAGAGAA	ATCC 700802
	vanBR	GCATTTGCCATGCAAAACC	
*tet(L)*	tetLF	GGGTAAAGCATTTGGTCTTATTGG	RBH200523
	tetLR	ATCGCTGGACCGACTCCTT	
*tet(M)*	tetMF	GCAGAATATACCATTCACATCGAAGT	RBH200535
	tetMR	AAACCAATGGAAGCCCAGAA	
*tet(S)*	tetSF	CCATTGATATCGAAGTACCTCCAA	RBH200535
	tetSR	AGGAAGTGGTGTTACAGATAAACCAA	
*gyr A*	gyrAF	CGGATGAACGAATTGGGTGTGA	ATCC 51559
	gyrAR	AATTTTACTCATACGTGCTT	
*pbp 5*	pbp5F	GTTCTGATCGAACATGAAGTTCAAA	ATCC 51559
	pbp5R	TGTGCCTTCGGATCGATTG	
*aac(6')-aph(2')*	acc-aphF	TCCTTACTTAATGACCGATGTACTCT	ATCC 700802
	acc-aphR	TCTTCGCTTTCGCCACTTTGA	

F^a ^forward primer, R^b ^reverse primer

#### Real-Time PCR

Each reaction contained 2 μl of DNA which was added to 18 μl of reaction master mix containing 10 μl of 2 × SYBRGreen^® ^PCR Mastermix (Invitrogen, Australia) and 0.25 μl of reverse and forward primers (20 μM stock, final concentration 0.5 μM). Cycling conditions were as follows: 50°C for 2 min, 95°C for 10 minutes, followed by 40 cycles of 95°C for 15 seconds, 60°C for 60 seconds, and a melting stage of 60°C-90°C. Each isolate was tested in duplicate. No Template Controls (NTCs) and previously characterized positive controls were used for each primer set as well.

### Mutation detection in the *gyrA *and *pbp5 *genes

All ciprofloxacin- and ampicillin-resistant and intermediate-resistant isolates were screened for gene mutations. The *gyrA *and *pbp5 *genes were amplified and sequenced. Primers used were: 5'CGGGATGAACGAATTGGGTGTGA3'and 5' AATTTTACTCATACGTGCTTCGG 3' (*gyrA *forward and reverse respectively); and 5' CGGGATCTCACAAGAAGAT 3'and 5' TTATTGATAATTTTGGTT 3' (*pbp5 *forward and reverse respectively) [34-36]. Sequencing reactions were prepared as for the SNP validation step described above. Sequence data was analysed using Chromas (version 1.43, Technelysium, Tewantin, Australia) and Vector NTI (version 11, Invitrogen, Australia) software programs.

## Results and Discussion

The poor microbiological quality of recreational waters is a global issue [37,38]. There is a great need to rapidly and accurately determine human faecal contamination of recreational waters. We applied a SNP genotyping method to water samples collected from the Coomera River, South East Queensland, Australia, to determine the distribution and diversity of *E. faecalis *and *E. faecium *strains and establish the antibiotic profiles associated with different SNP profiles.

### Total enterococccal counts in the Coomera River, over a two year period

Enumeration of enterococcal strains was performed at each of the six sampling sites along the Coomera River, and these counts were compared to the single-sample advisory limit specified by the US Environmental Protection Agency (USEPA) and the Australian NHMRC Guidelines for water quality assessment. Previous studies have found that the concentration of faecal indicator bacteria in surface waters is influenced by storm water runoff and can increase dramatically during rainfall events in comparison to baseline conditions [39-42]. Similarly, we found an increase in the number of enterococci at three of the sampling sites after rainfall events (August 2008 and March 2009). There was a substantial increase in enterococcal colony counts at Jabiru Island (C4), Paradise Point (C5) and Coombabah (C6) after rainfall events. These findings were confirmed by the Mann-Whitney test which showed that enterococcal counts after rainfall events differ significantly between the different locations; C4-C5 (p = 0.004) compared to C1-C3 (p = 0.029), (additional file 1). These counts were well above the USEPA recommended level (61 cfu/100 ml). According to the Australian NHMRC Guidelines these locations are categorised into the microbial water quality assessment category B (41-200 cfu/100 ml), except for Jabiru Island (March 2009), which was category C (201-500 cfu/100 ml). Category B indicates a 1-5% gastrointestinal illness risk and category C indicates a 5-10% gastrointestinal illness risk. In contrast, even though counts for the other sampling points, Marina (C1), Sanctuary Cove (C2) and Santa Barbara (C3) increased after rainfall, they were within the acceptable range for enterococci in fresh recreational water. Table 3 lists the total enterococcal counts (cfu/ml) for each of the sampling sites across the different sampling times.

**Table 3:** Total enterococcal counts at different sampling points at different sampling times

Site marked on the map	Site name	**Average concentration of enterococci cfu**^**a**^/**100 mL, ± STD**^**b**^			
		
		May-08	**Aug-08**^**C**^	**Mar-09**^**C**^	Jul-09
C1	Coomera marina	0 (0)	3 ± 1.41 (3)^d^	21.5 ± 2.12 (20)	4.5 ± 0.71 (5)
C2	Santa Barbara	0 (0)	2.5 ± 0.70 (3)	3.5 ± 0.71 (4)	0 (0)
C3	Sanctuary Cove	1.5 ± 0.7 (1)	32.5 ± 2.1 (20)	8.5 ± 2.12 (9)	3 ± 0 (3)
C4	Jabiru Island	5.5 ± 0.7 (6)	78 ± 4.2 (25)	230 ± 28.28 (30)	2.5 ± 0.70 (3)
C5	Paradise Point	9 ± 1.4 (10)	185 ± 7.0 (25)	160 ± 14.14 (25)	22 ± 1.41 (20)
C6	Coombabah	7.5 ± 0.71 (8)	165 ± 7.0 (25)	125 ± 7.07 (25)	4 ± 0 (4)

^a ^colony forming units

^b ^standard deviation

^c ^samples collected after rainfall event

^d ^number of isolates analysed

These high counts can be explained by the transportation of faecal indicator bacteria by storm water run-off [39-41] and soil leaching [37] immediately after a rainfall event. Storm water run-off occurs when rainfall is unable to infiltrate the soil surface (after soil saturation) and runs over land to transport soil particles, faecal and associated bacteria [39,42]. Increased urbanization and land usage changes in the South-East region of Queensland, has had an adverse impact on the quality of natural water resources [43]. One potential source of bacterial contamination may be the accidental sewage discharge from a large number of yachts and houseboats owned by residents with boat-moorings in these waterways. Furthermore, it is speculated that higher enterococcal counts at Jabiru Island (C4), Paradise Point (C5) and Coombabah (C6), compared, to Marina (C1), Sanctuary Cove (C2) and Santa Barbara (C3) may be due to their physical locations along the Coomera River and the impact of their surroundings. At Jabiru Island (C4), there is sand mine and the water is turbid particularly during rainfall periods. Previous studies have demonstrated that indicator organisms attach to sand particles [44]. Soil resuspension can be enhanced by rainfall, and as a result, higher enterococcal counts are possible. Paradise Point (C5) is a highly populated area and is used for bathing primarily. At Coombabah (C6), there is a waste-water treatment plant near the sampling site, and during rainfall periods, it is possible that there is a mixing of the treatment plant effluent with surrounding water bodies which contributes to high enterococcal counts. In addition, sampling sites C4-C6 are located at the lower reaches of the Coomera River, where enterococci can accumulate from the upstream regions of the river.

### The diversity and the distribution of *E. faecalis *and *E. faecium *SNP profiles in the Coomera River

It is more important to focus on *E. faecalis *and *E. faecium *rather than the total enterococcal count as they pose a definite human health risk and are the predominant enterococcal species in human faeces and sewage. In total, 55 *E. faecalis *and 47 *E. faecium *strains were isolated from six different sampling sites along the Coomera River. In this study, we applied a recently developed SNP genotyping method to the Coomera River to determine the diversity of *E. faecalis *and *E. faecium *genotypes. This method represents an efficient means of classifying *E. faecalis *and *E. faecium *into groups that are concordant with their population structure [29]. For the purpose of clarity, we define the SNP profiles into two main groups. The first group is the human-specific SNP profile group; these profiles are associated with enterococcal strains that originate from human samples only, as defined by the MLST database, as well as our previous study [27]. The second group is the human-related SNP profile group; these profiles are associated with enterococcal strains that originate from mixed sources (human and animal) according to the MLST database, but we found these profiles for enterococcal isolates from human specimens as well [27].

**Figure 2:**
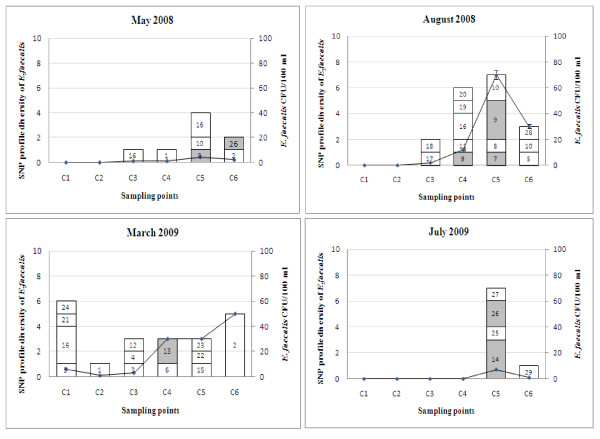
**Distribution of the *E. faecalis *SNP genotypes at six sampling points over a two year period**. Shaded areas indicate common SNP profiles for both human and water isolates. *E. faecalis *cfu/ml is indicated by the line graph. Total number of *E. faecalis *analysed during each sampling; May 2008 - 8 isolates, August 2008 - 18 isolates, March 2009 - 21 isolates and July 2009 - 8 isolates.

**Figure 3:**
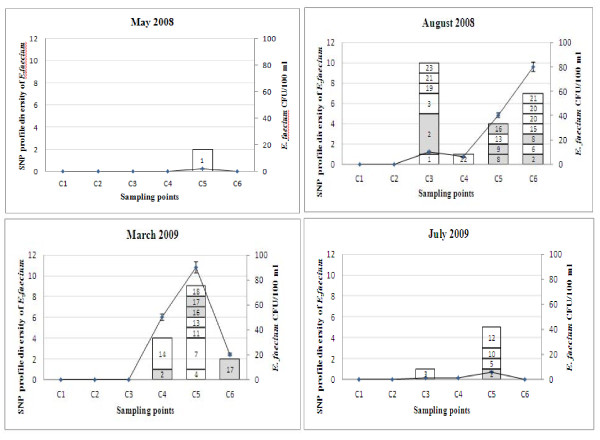
**Distribution of the *E. faecium *SNP genotypes at six sampling points over a two year period**. Shaded areas indicate common SNP profiles for both human and water isolates. *E. faecium *cfu/ml is indicated by the line graph. Total number of *E. faecium *analysed during each sampling; May 2008 - 2 isolates, August 2008 - 23 isolates, March 2009 - 16 isolates and July 2009 - 6 isolates.

The SNP profiles of the Coomera enterococcal strains were compared to known human-related and human-specific SNP profiles described previously [29]. SNP profiles were validated by gene sequencing using MLST primers for *E. faecalis *and *E. faecium*. Enterococcal strains with new SNP profiles (3 and 10 profiles for *E. faecalis *and *E. faecium *respectively) were also sequenced, and added to the MLST database (Tables 4 and 5). The Coomera isolates were grouped into 29 and 23 SNP profiles for *E. faecalis *and *E. faecium *respectively (Tables 4 and 5). These results confirm that the enterococcal population in the Coomera River is diverse. Figures 2 and 3 illustrate the distribution of these SNP profiles at all sampling points over the two year study period. In addition, we found that both *E. faecalis *and *E. faecium *populations were more diverse during rainfall periods (August 2008 and March 2009).

**Table 4:** SNP and antibiotic resistance gene profiles of *E. faecalis *isolates and their corresponding Sequence Types (STs)

SNP profile	SNP ID	No of isolates	Antibiotic resistance [Identified genetic determinants]	Corresponding sequence Types (STs) for SNP profiles in MLST
ACCAAACC	1	2	Cip ^IR ^[*gyrA*]; Tet ^R ^[ *tetM*]	ST4, ST22, ST32, ST129, ST202
**ACCAAACT**	2	5	Tet ^R ^[*tetM*]	ST274
**ACCAAACT**	2	1	Gen ^IR ^[*aac(6')-aph(2'*)] Tet ^R ^[ *tetM*]	
ACCGAGCT	3	1	No antibiotic resistance detected	ST277
ACCGAGTT	4	1	No antibiotic resistance detected	ST123
ACCGTGCC	5	2	Gen ^IR ^[*aac(6')-aph(2'*)]	ST76
ACTAAGCT	6	1	Gen ^R ^[*aac(6')-aph(2'*)]	ST278
**ACTATGCC**^a^	**7**	1	Gen ^R ^[*aac(6')-aph(2'*)]	ST79, ST82
ACTGTGTC	8	1	No antibiotic resistance detected	ST414^N^
**ATCAAACC**	**9**	3	Gen ^R ^[*aac(6')-aph(2'*)]; Cip ^IR ^[*gyrA*]	ST5, ST21, ST46, ST50, ST70, ST145, ST152, ST157
**ATCAAACC**	**9**	1	Gen ^IR ^[*aac(6')-aph(2'*)]	
**ATCAAACC**	**9**	1	Gen ^R ^[*aac(6')-aph(2'*)]	
ATCGTGCC	10	4	Gen ^IR ^[*aac(6')-aph(2'*)]	ST143
ATCGTGTT	11	1	Gen ^R ^[*aac(6')-aph(2'*)]	ST230
ATTAAACC	12	1	Cip ^IR ^[*gyrA*]	ST255
**ATTAAGCT**	**13**	2	Gen ^R ^[*aac(6')-aph(2'*)]; Cip ^IR ^[*gyrA*]	ST139, ST181, ST183, ST241
**ATTATGCC**	**14**	3	Gen ^R ^[*aac(6')-aph(2'*)]; Cip ^IR ^[*gyrA*]	ST170
GCCAAACT	15	1	No antibiotic resistance detected	ST410
**GCCATGCT**	16	1	Gen ^R ^[*aac(6')-aph(2'*)]; Cip ^IR ^[*gyrA*]	ST81, ST164
**GCCATGCT**	16	7	Gen ^R ^[*aac(6')-aph(2'*)]	
GCCGTACC	17	1	No antibiotic resistance detected	ST110
GCCGTGCC	18	1	No antibiotic resistance detected	ST27, ST124
GCCGTGCT	19	1	Gen ^R [^*aac(6')-aph(2'*)]	ST418^N^
GCCGTGTC	20	1	No antibiotic resistance detected	ST86
GCTATACC	21	1	Gen ^R ^[*aac(6')-aph(2'*)]	ST39, ST45, ST69, ST96, ST116
GCTGTACC	22	1	No antibiotic resistance detected	ST260, ST396
GCTGTGTT	23	1	No antibiotic resistance detected	ST201
ATTAAGCC	24	1	Gen ^IR [^*aac(6')-aph(2'*)]	ST419^N^
GTCGTATT	25	1	Gen ^R ^[*aac(6')-aph(2'*)]	ST125, ST165, ST167
**GTCGTGTT**	**26**	2	Gen ^R ^[*aac(6')-aph(2'*)]; Cip ^IR ^[*gyrA*]	ST36, ST118, ST180
**GTCGTGTT**	**26**	1	Cip ^IR ^[*gyrA*]	
GTTATGCC	27	1	Gen ^R ^[*aac(6')-aph(2'*)]	ST108, ST122
**GTTGAGTC**	**28**	1	Gen ^R ^[*aac(6')-aph(2'*)]; Cip ^IR ^[*gyrA*]	ST64, ST101, ST161, ST205
GTTATGCT	29	1	No antibiotic resistance detected	ST175

^a^SNP profiles which were present in hospital isolates are bold and underlined

SNP profiles in bold text are isolates with the same SNP profiles but with different antibiotic resistant gene profiles.

Amp; ampicillin, Cip; ciprofloxacin, Gen; gentamicin, Tet; tetracycline,

^R ^Resistant, ^IR ^Intermediate resistant

^N ^New sequence types identified in the present study

**Table 5:** SNP and antibiotic resistance gene profiles of *E. faecium *isolates and their corresponding Sequence types (STs)

SNP Profile	SNP ID	No. of Isolates	Antibiotic resistance [Identified genetic determinants]	Corresponding sequence Types (STs) for SNP profiles in MLST
AAACTTTC	1	3	No antibiotic resistance detected	ST 544, ST 583
**AACCCTTC**^**a**^	**2**	1	Amp ^R ^[*pbp5*]^m^; Tet ^R ^[*tetM*]	ST 602 ^N^
**AACCCTTC**^**a**^	**2**	3	Cip ^IR ^[*gyrA*]	
**AACCCTTC**^**a**^	**2**	2	No antibiotic resistance detected	
**AATCCTTC**	3	1	Gen ^IR ^[*aac(6')-aph(2'*)]; Cip ^IR ^[*gyrA*]	ST8, ST9, ST58, ST134, ST194, ST198, ST237, ST244, ST248,
**AATCCTTC**	3	2	No antibiotic resistance detected	ST259, ST266, ST298, ST309, ST370, ST402, ST425,
AATCTTTC	4	1	No antibiotic resistance detected	ST40, ST100, ST163, ST211, ST221, ST223, ST226,
AGCCCCTC	5	1	Gen ^IR ^[*aac(6')-aph(2'*)]	ST 607 ^N^
AGCCCTCT	6	1	No antibiotic resistance detected	ST508
**AGCCCTTT**	7	2	Cip ^IR ^[*gyrA*]	ST 603 ^N^
**AGCCCTTT**	7	1	No antibiotic resistance detected	
**AGCCTTTC**	**8**	2	No antibiotic resistance detected	ST 604 ^N^
**AGCTCTCC**	**9**	2	Gen ^IR ^[*aac(6')-aph(2'*)];Cip ^R ^[*gyrA*]^m^;Amp ^R ^[*pbp5*]^m^	ST260, ST262, ST273, ST322
AGTCCTTC	10	2	Gen ^IR ^[*aac(6')-aph(2'*)]; Cip ^R ^[*gyrA*]^m^	ST13, ST14, ST48, ST79, ST82, ST120, ST157, ST195, ST200, ST241
			Tet ^R ^[*tetL & S*]	ST242, ST310, ST311,
AGTCCTTT	11	1	Gen ^IR ^[*aac(6')-aph(2'*)]; Cip ^IR ^[*gyrA*]	ST15, ST70
AGTCTTTT	12	2	Cip ^IR ^[*gyrA*]	ST 605 ^N^
**GACCCTCC**	13	1	Gen ^IR ^[*aac(6')-aph(2'*)]	ST 608 ^N^
**GACCCTCC**	13	1	Cip ^IR ^[*gyrA*]	
**GACCCTTT**	14	1	Cip ^IR ^[*gyrA*]; Tet ^R ^[*tetL & M*]	ST 609 ^N^
**GACCCTTT**	14	2	Tet ^R ^[*tetL,M & S*]	
GATCCTTC	15	1	Gen ^IR ^[*aac(6')-aph(2'*)]	ST 610 ^N^
**GGCCCCCC**	**16**	2	Cip ^IR ^[*gyrA*]	ST501, ST511, ST516, ST518, ST521, ST522, ST529, ST530, ST565, ST588, ST592, ST599

**GGCCCTCC**	**17**	3	Amp ^R ^[*pbp5*]^m^; Tet ^IR ^[*tetL & tetM*]	ST162
GGCCCTTC	18	2	Cip ^IR ^[*gyrA*]	ST 611 ^N^
GGCCTCCC	19	1	No antibiotic resistance detected	ST 606 ^N^
GGTCCCCC	20	2	Cip ^IR ^[*gyrA*]	ST22, ST23, ST24, ST27, ST28, ST33, ST36, ST55, ST59, ST106,
				ST111, ST119, ST122, ST131, ST136, ST159, ST214, ST263, ST269,
				ST270, ST315, ST372, ST379, ST422, ST435
GGTCCTCC	21	1	Cip ^R ^[*gyrA*]^m^	ST1, ST2, ST3, ST4, ST32, ST34, ST35, ST41, ST43, ST72,
				ST87, ST90, ST104, ST109, ST128, ST139, ST146, ST215, ST250, ST253
				ST257, ST291, ST292, ST330, ST426, ST427,
GGTCCTTT	22	1	Cip ^IR ^[*gyrA*]	ST6, ST88, ST149, ST170, ST247, ST255, ST354
GGTCCTTC	23	2	No antibiotic resistance detected	ST7, ST21, ST25, ST26, ST29, ST37, ST57, ST66, ST68, ST83,
				ST89,ST97,ST99,ST112,ST113,ST115,ST124, ST129, ST143, ST158,
				ST160, ST176, ST183, ST210, ST236, ST243, ST245, ST246, ST251,
				ST265, ST271, ST272, ST277, ST281, ST284, ST297, ST358, ST381,
				ST384, ST395, ST401, ST418, ST433, ST437,

^a^SNP profiles which were present in hospital isolates are bold and underlined

SNP profiles in bold text are isolates with the same SNP profiles but with different antibiotic resistant gene profiles.

Amp; ampicillin, Cip; ciprofloxacin, Gen; gentamicin, Tet; tetracycline,

^R ^Resistant, ^IR ^Intermediate resistant, ^m ^mutation

^N ^New sequence types identified in the present study

Of all the SNP profiles described in this study, six SNP profiles ACTATGCC, ATCAAACC, ATTAAGCT, ATTATGCC, GTCGTGTT and GTTGAGTC (ID no: 7, 9, 13, 14, 26 & 28) of *E. faecalis *and five *E. faecium *SNP profiles AACCCTTC, AGCCTTTC, AGCTCTCC, GGCCCCCC and GGCCCTCC (ID no: 2, 8, 9, 16 & 17) have previously been described and distributed amongst human strains in Brisbane, Australia [29]. SNP profiles 7, 9, 13, 14 & 26 of *E. faecalis *and 16 of *E. faecium *have previously been found to correspond to not only human *E. faecalis *and *E. faecium *strains listed in the MLST database, but these SNP profiles also include strains originating from other sources such as animals. These SNP profiles are therefore classified as human-related SNP profiles [29]. *E. faecalis *SNP profile 28 and *E. faecium *SNP profiles 2, 8, 9 and 17 are found only in humans and classified as human-specific. eBURST analysis of both the *E. faecalis *and *E. faecium *MLST database, which now include the new STs found in this study, are included as additional file 2. The new *E. faecium *STs, ST602 (SNP profile 2) and ST604 (SNP profile 8), found in this study are human-specific and not related to the major clonal complex-17 (CC17), as shown in the eBURST diagram (Additional file 2). A very important finding of this study was the isolation of *E. faecium *strains (4.25%) with SNP profile AGCTCTCC (ID no. 9) from water, as we have previously demonstrated that this is a human-specific SNP profile which represents a major clonal complex-17 (CC17) of *E. faecium *strains that cause the majority of hospital outbreaks and clinical infections across five continents [45,46]. Of major concern is the fact that the majority of the members of this cluster are vancomycin-resistant and CC17 strains are generally resistant to ampicillin and carry genes for putative virulence factors, such as *esp *[47]. The dissemination of these types of strains in natural waterways is of concern and further investigations are warranted to establish the genetic similarity between water *E. faecium *strains and those originating from clinical sources.

Overall, these human-related and human-specific enterococcal SNP profiles were found at Jabiru Island (SNP ID 9 &13 of *E. faecalis *and SNP ID 2 of *E. faecium*) and Coombabah (SNP ID 28 of *E. faecalis *and SNP ID 2, 8 and 17 of *E. faecium*) after rainfall events, where the total enterococcal count was above the USEPA acceptable level. A likely reason for this occurrence is the terrestrial run-off during high rainfall. In contrast, at Paradise Point, the human-related *E. faecalis *and *E. faecium *SNP profiles were detected irrespective of rainfall. SNP profiles 7, 9, 14 & 26 of *E. faecalis*, and SNP profiles 2, 8, 9, 16 and 17 of *E. faecium *were found at Paradise Point. Furthermore, SNP profiles 9, 14 and 26 of *E. faecalis *and SNP profile 2 of *E. faecium *were found in the absence of rain. In comparison to other sites, Paradise Point had the highest number of human-related and human-specific SNP profiles. Paradise Point is primarily used for public bathing, and therefore the presence of these human-related and human-specific enterococcal SNP profiles indicates human faecal contamination of this area.

### Antibiotic resistance profiles related to SNP profiles

Tables 4 and 5 summarize the antibiotic resistance profiles for the *E. faecalis *and *E. faecium *strains tested in this study. Disc susceptibility results are included in additional files 3 and 4. The outcome of the antimicrobial disc susceptibility tests followed by PCR, revealed that 81.8% of *E. faecalis *SNP profiles and 70.21% of *E. faecium *SNP profiles were associated with antibiotic resistance. The highest percentage of antibiotic resistant *E. faecalis *was found at Paradise Point (C5) 37.7% followed by Coombabah (C6) 22.2%, Jabiru Island (C4) 19.1%, Marina (C1) 15.5%, Santa Barbara (C3) 4.4% and Sanctuary Cove (C2) 2.2%. No antibiotic resistant *E. faecium *strains were found at Marina (C1) and Sanctuary Cove (C2). The highest percentage of antibiotic resistant *E. faecium *was found at Paradise Point (C5) 51.5% followed by Coombabah (C6) 21.2%, Jabiru Island (C4) 15.1% and Santa Barbara (C3) 12.1%. Phenotypic and genotypic antibiotic resistance profiles of *E. faecalis *and *E. faecium *at individual sampling sites are listed in additional files 5 and 6.

Gentamicin resistance was more prevalent in *E. faecalis *(47% resistant and 16% intermediate resistant) and these strains contained the *aac*(6')-*aph*(2') gene. Whereas ciprofloxacin resistance is more common in *E. faecium *(12.7% resistant and 36.2% intermediate-resistant). According to previous studies, one of the factors used to determine ciprofloxacin resistance is the association with mutations in the DNA gyrase genes [34]. The sequencing results revealed that there were no mutations detected in *gyrA *gene of intermediate resistant strains, however, amino acid changes were detected in five *E. faecium *isolates that were disc-resistant to ciprofloxacin. Amino acid changes at position 83 (serine to arginine) were found in two isolates belonging to SNP ID 9, whereas the remaining three isolates, belonging to SNP ID 10 and 21 had an amino acid change at position 87 (glutamate to lysine). According to previous studies, glutamate at position 87 can also be replaced by glycine in ciprofloxacin-resistant isolates, but this was not detected in our environmental isolates [34]. Tetracycline resistance was less common among *E. faecalis *(14%) and *E. faecium *(12.7%) strains. Of these, the *tet*(L) and *tet*(M) genes were the predominant genetic determinants. This finding is consistent with previous studies [48]. Ampicillin resistance was found in only six *E. faecium *strains. Ampicillin resistance was observed in both multi-drug resistant strains and in human-related strains. Previous studies have shown an amino acid substitution in ampicillin-resistant enterococci. Potentially significant mutations that confer ampicillin resistance are methionine to alanine substitution at position 485, an additional serine at position 466, and replacement of a polar amino acid with a non-polar one (alanine or isoleucine) at position 558, 562, or 574. A glutamate to valine substitution at position 629 has also been associated with ampicillin resistance [49]. In the present study, an ampicillin-resistant *E. faecium *isolate with SNP ID 2 had alanine at position 485 and all the other ampicillin- resistant *E. faecium *strains had valine at position 629 (SNP ID 9 and 17). Vancomycin resistance was not detected in any of the environmental isolates tested. Multi-antibiotic resistance was found in both *E. faecalis *(27%) and *E. faecium *(22%). Of these isolates, all *E. faecalis *harboured only two resistance genes. Eight *E. faecium *isolates with SNP IDs 9, 10 and 17 harbored more than three antibiotic resistance genes. However, it is interesting to note that SNP ID no. 9, which represents CC17, had multi-antibiotic resistance and contained the *aac*(6')-*aph*(2') gene and had mutations in the *gyrA *and *pbp*5 genes. This supports the notion that members of CC17 are reservoirs of multidrug-resistance genes in the environment [50]. Hospital SNP profiles for both *E. faecalis *and *E. faecium*. (Bold and underlined text in Tables 4 and 5), were antibiotic-resistant by both disc and PCR methods.

The SNP profiles in bold text in Tables 4 and 5 highlight the isolates that had the same SNP profile but had different antibiotic-resistant gene profiles which resulted in sub-dividing the SNP profiles. A possible explanation for this is that the SNPs interrogated by our method, are located in housekeeping genes, which are considered conservative, whereas, antibiotic resistance determinants are "mobile" except for the *gyrA *and *pbp5 *genes. *E. faecalis *SNP IDs 2, 16 and 26 and *E. faecium *SNP IDs 3, 7, 13 and 14 were sub-divided into two groups. In addition, *E. faecalis *isolates with SNP ID 9 and *E. faecium *SNP ID 2 can be can be sub-divided in to three groups. These antibiotic-resistant profiles can be used to increase the resolving power of the SNP typing method.

## Conclusion

This study describes the prevalence and distribution of *E. faecalis *and *E. faecium *SNP profile genotypes in the Coomera River. The SNP genotyping method demonstrates a high diversity in the *E. faecalis *and *E. faecium *population in the Coomera River. In addition, at three sampling sites (Jabiru Island, Paradise Point and Coombabah), the enterococcal counts were above the USEPA acceptable levels after rainfall events. According to the Australian NHMRC Guidelines these sampling sites are category B and C areas according to the microbial water quality assessment (after rainfall), with category B indicating a 1-5% gastrointestinal illness risk and category C indicating a 5-10% gastrointestinal illness risk. We have also demonstrated the application of the SNP genotyping method to identify both human-related and human-specific *E. faecium *and *E. faecalis *strains in environmental water sources. This method shows promise as a rapid and robust test to determine human faecal contamination of environmental water sources. Some strains were antibiotic resistant and these antibiotic resistant profiles can be used as binary markers to increase the discriminatory power of the SNP genotyping method.

## Authors' contributions

IUR performed the experiments, analysed the data and drafted the manuscript. MH assisted with the drafting of the manuscript. FH conceived the study, contributed to the experimental design, co-ordinated data analysis and assisted with the drafting of the manuscript. All authors have read and approved the final manuscript.

## Supplementary Material

Additional file 1 **Statistical analysis Mann-Whitney test**. This test was performed to determine whether there was a significant increase in total enterococcal counts (cfu/ml) at each location after rainfall events. Click here for file

Additional file 2 **e-BURST diagrams of both *E. faecium *and *E. faecalis *MLST databases**. Each diagram shows the new STs found in the present study compared to all the STs currently listed in both databases. Click here for file

Additional file 3 **Disc susceptibility test results for *E. faecalis***. This table lists the antibiotic disc susceptibility profiles for all *E. faecalis *isolates tested in this study. Click here for file

Additional file 4 **Disc susceptibility test results for *E. faecium***. This table lists the antibiotic disc susceptibility profiles for all *E. faecium *isolates tested in this study. Click here for file

Additional file 5 **Phenotypic and genotypic antibiotic resistance profiles of *E. faecalis *isolated at each site**. Antibiotic resistance profiles together with the *E. faecalis *SNP profiles of strains isolated at all the sampling sites are listed here. Click here for file

Additional file 6 **Phenotypic and genotypic antibiotic resistance profiles of *E. faecium *isolated at each site**. Antibiotic resistance profiles together with the *E. faecium *SNP profiles of strains isolated at all the sampling sites are listed here. Click here for file
