# Supplementary material for: SNP diversity of Enterococcus faecalis and Enterococcus faecium in a South East Queensland waterway, Australia, and associated antibiotic resistance gene profiles
Source: BMC Microbiol. 2011 Sep 12;11:201. doi: 10.1186/1471-2180-11-201 (PMC3179957; doi:10.1186/1471-2180-11-201)

**Additional file 1- Statistical analysis Mann-Whitney test**

Statistical analysis supplementary data

Mann-Whitney test for C1-C3

Data arrangement

| cfu/ml without rainfall   | 1 | 0 |  | | --- | --- | --- | | 1 | 0 | | 1 | 1.5 | | 1 | 4.5 | | 1 | 0 | | 1 | 3 | | cfu/ml with rainfall | 2 | 3 | | 2 | 2.5 | | 2 | 32.5 | | 2 | 21.5 | | 2 | 3.5 | | 2 | 8.5 | |  |
| --- | --- | --- | --- | --- | --- | --- | --- | --- | --- | --- | --- | --- | --- | --- | --- | --- | --- | --- | --- | --- | --- | --- | --- | --- | --- | --- | --- |
| 1-Enterococci cfu/100ml without rainfall  2-Enterococci cfu/100ml with rainfall |  |
|  |  |
|  |  |
| 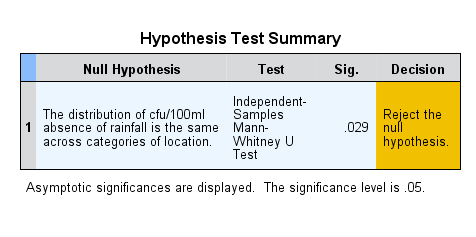 |  |
|  |  |
|  |  |
|  |  |
|  |  |
|  |  |
|  |  |
|  |  |

Mann-Whitney test for C4-C6

Data arrangement

| cfu/ml without rainfall | 1 | 5.5 |
| --- | --- | --- |
|  | 1 | 9 |
|  | 1 | 7.5 |
|  | 1 | 2.5 |
|  | 1 | 22 |
|  | 1 | 4 |
| cfu/ml with rainfall | 2 | 78 |
|  | 2 | 185 |
|  | 2 | 165 |
|  | 2 | 230 |
|  | 2 | 160 |
|  | 2 | 125 |

1-Enterococci cfu/100ml without rainfall

2-Enterococci cfu/100ml with rainfall


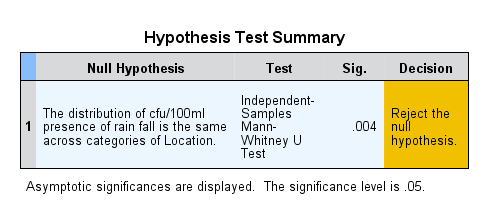

Supplement: Additional file 1 — Statistical analysis Mann-Whitney test. This test was performed to determine whether there was a significant increase in total enterococcal counts (cfu/ml) at each location after rainfall events. [file 1471-2180-11-201-S1.DOC]
