# Supplementary material for: SNP diversity of Enterococcus faecalis and Enterococcus faecium in a South East Queensland waterway, Australia, and associated antibiotic resistance gene profiles
Source: BMC Microbiol. 2011 Sep 12;11:201. doi: 10.1186/1471-2180-11-201 (PMC3179957; doi:10.1186/1471-2180-11-201)

**Additional file - 2**

**e-BURST diagram representing the *E. faecium* STs of the present study together with all the STs currently listed in the MLST database. New STs found in this study are highlighted in blue and in some cases, inidicated by a red line.**


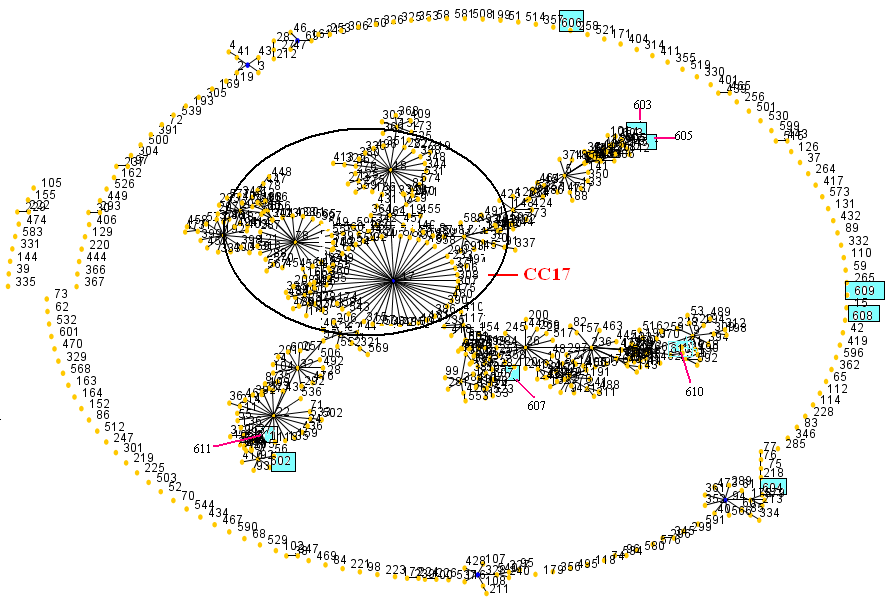


**e-BURST diagram representing the *E. faecalis* STs of the present study together with all the STs currently listed in the MLST database. New STs found in this study are highlighted in blue.**


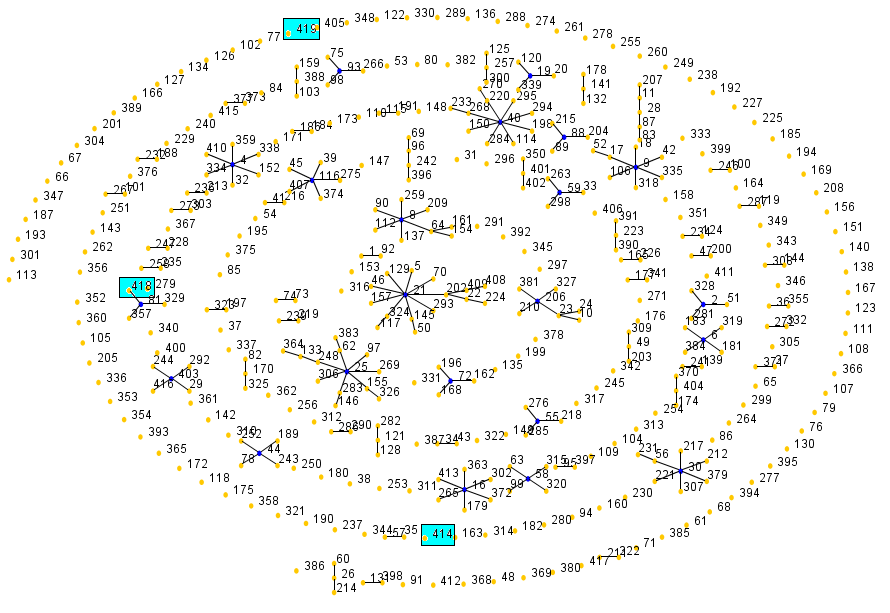

Supplement: Additional file 2 — e-BURST diagrams of both E. faecium and E. faecalis MLST databases. Each diagram shows the new STs found in the present study compared to all the STs currently listed in both databases. [file 1471-2180-11-201-S2.DOC]
