# Supplementary material for: SNP diversity of Enterococcus faecalis and Enterococcus faecium in a South East Queensland waterway, Australia, and associated antibiotic resistance gene profiles
Source: BMC Microbiol. 2011 Sep 12;11:201. doi: 10.1186/1471-2180-11-201 (PMC3179957; doi:10.1186/1471-2180-11-201)
Supplement: Additional file 3 — Disc susceptibility test results for E. faecalis. This table lists the antibiotic disc susceptibility profiles for all E. faecalis isolates tested in this study. [file 1471-2180-11-201-S3.DOC]

**Additional file 3- Disc susceptibility test results for *E. faecalis***

|  |  |  | ***E. faecalis* antibiotic disc susceptibility profiles** | | | | | | | | | | | | | | |
| --- | --- | --- | --- | --- | --- | --- | --- | --- | --- | --- | --- | --- | --- | --- | --- | --- | --- |
|  |  |  | **teracycline** | | | **ciprofloxacin** | | | **gentamicin** | | | **ampicillin** | | | **vancomycin** | | |
| **Sample No** | **SNP PROFILE** | **SNP ID** | **R** | **IR** | **S** | **R** | **IR** | **S** | **R** | **IR** | **S** | **R** | **IR** | **S** | **R** | **IR** | **S** |
| 1/C4/1 | ACCAAACC | 1 | + | - | - | - | + | - | - | - | + | - | - | + | - | - | + |
| 3/C2/3 | ACCAAACC | 1 | + | - | - | - | + | - | - | - | + | - | - | + | - | - | + |
| 1/C6/1 | ACCAAACT | 2 | + | - | - | - | - | + | - | - | + | - | - | + | - | - | + |
| 3/C6/3 | ACCAAACT | 2 | + | - | - | - | - | + | - | - | + | - | - | + | - | - | + |
| 3/C6/11 | ACCAAACT | 2 | + | - | - | - | - | + | - | + | - | - | - | + | - | - | + |
| 3/C6/12 | ACCAAACT | 2 | + | - | - | - | - | + | - | - | + | - | - | + | - | - | + |
| 3/C6/13 | ACCAAACT | 2 | + | - | - | - | - | + | - | - | + | - | - | + | - | - | + |
| 3/C6/14 | ACCAAACT | 2 | + | - | - | - | - | + | - | - | + | - | - | + | - | - | + |
| 3/C3/1 | ACCGAGCT | 3 | - | - | + | - | - | + | - | - | + | - | - | + | - | - | + |
| 3/C3/4 | ACCGAGTT | 4 | - | - | + | - | - | + | - | - | + | - | - | + | - | - | + |
| 2/C6/19 | ACCGTGCC | 5 | - | - | + | - | - | + | - | + | - | - | - | + | - | - | + |
| 3/C1/2 | ACCGTGCC | 5 | - | - | + | - | - | + | - | + | - | - | - | + | - | - | + |
| 3/C4/3 | ACTAAGCT | 6 | - | - | + | - | - | + | + | - | - | - | - | + | - | - | + |
| 2/C5/5 | ACTATGCC | 7 | - | - | + | - | - | + | + | - | - | - | - | + | - | - | + |
| 2/C5/4 | ACTGTGTC | 8 | - | - | + | - | - | + | - | - | + | - | - | + | - | - | + |
| 1/C5/3 | ATCAAACC | 9 | - | - | + | - | + | - | + | - | - | - | - | + | - | - | + |
| 2/C4/10 | ATCAAACC | 9 | - | - | + | - | - | + | + | - | - | - | - | + | - | - | + |
| 2/C5/3 | ATCAAACC | 9 | - | - | + | - | + | - | + | - | - | - | - | + | - | - | + |
| 2/C5/11 | ATCAAACC | 9 | - | - | + | - | + | - | + | - | - | - | - | + | - | - | + |
| 2/C5/18 | ATCAAACC | 9 | - | - | + | - | - | + | - | + | - | - | - | + | - | - | + |
| 1/C5/4 | ATCGTGCC | 10 | - | - | + | - | - | + | - | + | - | - | - | + | - | - | + |
| 2/C5/6 | ATCGTGCC | 10 | - | - | + | - | - | + | - | + | - | - | - | + | - | - | + |
| 2/C5/9 | ATCGTGCC | 10 | - | - | + | - | - | + | - | + | - | - | - | + | - | - | + |
| 2/C6/9 | ATCGTGCC | 10 | - | - | + | - | - | + | - | + | - | - | - | + | - | - | + |
| 2/C4/8 | ATCGTGTT | 11 | - | - | + | - | - | + | + | - | - | - | - | + | - | - | + |
| 3/C3/8 | ATTAAACC | 12 | - | - | + | - | + | - | - | - | + | - | - | + | - | - | + |
| 3/C4/1 | ATTAAGCT | 13 | - | - | + | - | + | - | + | - | - | - | - | + | - | - | + |
| 3/C4/10 | ATTAAGCT | 13 | - | - | + | - | + | - | + | - | - | - | - | + | - | - | + |
| 4/C5/2 | ATTATGCC | 14 | - | - | + | - | + | - | + | - | - | - | - | + | - | - | + |
| 4/C5/5 | ATTATGCC | 14 | - | - | + | - | + | - | + | - | - | - | - | + | - | - | + |
| 4/C5/6 | ATTATGCC | 14 | - | - | + | - | + | - | + | - | - | - | - | + | - | - | + |
| 3/C5/8 | GCCAAACT | 15 | - | - | + | - | - | + | - | - | + | - | - | + | - | - | + |
| 1/C3/2 | GCCATGCT | 16 | - | - | + | - | - | + | + | - | - | - | - | + | - | - | + |
| 1/C5/1 | GCCATGCT | 16 | - | - | + | - | - | + | + | - | - | - | - | + | - | - | + |
| 1/C5/2 | GCCATGCT | 16 | - | - | + | - | - | + | + | - | - | - | - | + | - | - | + |
|  |  |  | ***E. faecalis* antibiotic disc susceptibility profiles** | | | | | | | | | | | | | | |
|  |  |  | **tetracycline** | | | **ciprofloxacin** | | | **gentamicin** | | | **ampicillin** | | | **vancomycin** | | |
| **Sample No** | **SNP PROFILE** | **SNP ID** | **R** | **IR** | **S** | **R** | **IR** | **S** | **R** | **IR** | **S** | **R** | **IR** | **S** | **R** | **IR** | **S** |
| 2/C4/1 | GCCATGCT | 16 | - | - | + | - | - | + | + | - | - | - | - | + | - | - | + |
| 2/C4/11 | GCCATGCT | 16 | - | - | + | - | - | + | + | - | - | - | - | + | - | - | + |
| 3/C1/3 | GCCATGCT | 16 | - | - | + | - | + | - | + | - | - | - | - | + | - | - | + |
| 3/C1/5 | GCCATGCT | 16 | - | - | + | - | - | + | + | - | - | - | - | + | - | - | + |
| 3/C1/9 | GCCATGCT | 16 | - | - | + | - | - | + | + | - | - | - | - | + | - | - | + |
| 2/C3/10 | GCCGTACC | 17 | - | - | + | - | - | + | - | - | + | - | - | + | - | - | + |
| 2/C3/11 | GCCGTGCC | 18 | - | - | + | - | - | + | - | - | + | - | - | + | - | - | + |
| 2/C4/3 | GCCGTGCT | 19 | - | - | + | - | - | + | + | - | - | - | - | + | - | - | + |
| 2/C4/5 | GCCGTGTC | 20 | - | - | + | - | - | + | - | - | + | - | - | + | - | - | + |
| 3/C1/4 | GCTATACC | 21 | - | - | + | - | - | + | + | - | - | - | - | + | - | - | + |
| 3/C5/1 | GCTGTACC | 22 | - | - | + | - | - | + | - | - | + | - | - | + | - | - | + |
| 3/C5/7 | GCTGTGTT | 23 | - | - | + | - | - | + | - | - | + | - | - | + | - | - | + |
| 3/C1/1 | ATTAAGCC | 24 | - | - | + | - | - | + | - | + | - | - | - | + | - | - | + |
| 4/C5/1 | GTCGTATT | 25 | - | - | + | - | - | + | + | - | - | - | - | + | - | - | + |
| 1/C6/2 | GTCGTGTT | 26 | - | - | + | - | + | - | + | - | - | - | - | + | - | - | + |
| 4/C5/7 | GTCGTGTT | 26 | - | - | + | - | + | - | + | - | - | - | - | + | - | - | + |
| 4/C5/8 | GTCGTGTT | 26 | - | - | + | - | + | - | - | - | + | - | - | + | - | - | + |
| 4/C5/4 | GTTATGCC | 27 | - | - | + | - | - | + | + | - | - | - | - | + | - | - | + |
| 2/C6/18 | GTTGAGTC | 28 | - | - | + | - | + | - | + | - | - | - | - | + | - | - | + |
| 4/C6/2 | GPTATGCT | 29 | - | - | + | - | - | + | - | - | + | - | - | + | - | - | + |

^R^  Resistant , ^IR^  Intermediate resistant, ^S^ Susceptible
