# Supplementary material for: SNP diversity of Enterococcus faecalis and Enterococcus faecium in a South East Queensland waterway, Australia, and associated antibiotic resistance gene profiles
Source: BMC Microbiol. 2011 Sep 12;11:201. doi: 10.1186/1471-2180-11-201 (PMC3179957; doi:10.1186/1471-2180-11-201)
Supplement: Additional file 4 — Disc susceptibility test results for E. faecium. This table lists the antibiotic disc susceptibility profiles for all E. faecium isolates tested in this study. [file 1471-2180-11-201-S4.DOC]

**Additional file 4 – Disk susceptibility test results for *E.faecium***

|  |  |  | ***E. faecium* antibiotic disc susceptibility profiles** | | | | | | | | | | | | | | |
| --- | --- | --- | --- | --- | --- | --- | --- | --- | --- | --- | --- | --- | --- | --- | --- | --- | --- |
|  |  |  | **tetracycline** | | | **ciprofloxacin** | | | **gentamicin** | | | **ampicillin** | | | **vancomycin** | | |
| **Sample No** | **SNP PROFILE** | **SNP ID** | **R** | **IR** | **S** | **R** | **IR** | **S** | **R** | **IR** | **S** | **R** | **IR** | **S** | **R** | **IR** | **S** |
| 1/C5/2 | AAACTCTC | 1 | - | - | + | - | - | + | - | - | + | - | - | - | - | - | + |
| 1/C5/4 | AAACTCTC | 1 | - | - | + | - | - | + | - | - | + | - | - | - | - | - | + |
| 2/C3/3 | AAACTCTC | 1 | - | - | + | - | - | + | - | - | + | - | - | - | - | - | + |
| 2/C3/4 | AACCCTTC | 2 | - | - | + | - | - | + | - | - | + | - | - | - | - | - | + |
| 2/C3/7 | AACCCTTC | 2 | - | - | + | - | + | - | - | - | + | - | - | - | - | - | + |
| 2/C3/8 | AACCCTTC | 2 | - | - | + | - | + | - | - | - | + | - | - | - | - | - | + |
| 2/C3/11 | AACCCTTC | 2 | - | - | + | - | - | + | - | - | + | - | - | - | - | - | + |
| 2/C6/10 | AACCCTTC | 2 | - | - | + | - | + | - | - | - | + | - | - | - | - | - | + |
| 3/C4/6 | AACCCTTC | 2 | + | - | - | - | - | + | - | - | + | + | - | - | - | - | + |
| 2/C3/1 | AATCCTTC | 3 | - | - | + | - | - | + | - | - | + | - | - | - | - | - | + |
| 2/C3/5 | AATCCTTC | 3 | - | - | + | - | - | + | - | - | + | - | - | - | - | - | + |
| 4/C3/1 | AATCCTTC | 3 | - | - | + | - | + | - | - | + | - | - | - | - | - | - | + |
| 3/C5/1 | AATCTTTC | 4 | - | - | + | - | - | + | - | - | + | - | - | - | - | - | + |
| 4/C5/5 | AGCCCCTC | 5 | - | - | + | - | - | + | - | + | - | - | - | - | - | - | + |
| 2/C6/15 | AGCCCTCT | 6 | - | - | + | - | - | + | - | - | + | - | - | - | - | - | + |
| 3/C5/6 | AGCCCTTT | 7 | - | - | + | - | + | - | - | - | + | - | - | - | - | - | + |
| 3/C5/9 | AGCCCTTT | 7 | - | - | + | - | + | - | - | - | + | - | - | - | - | - | + |
| 3/C5/10 | AGCCCTTT | 7 | - | - | + | - | - | + | - | - | + | - | - | - | - | - | + |
| 2/C5/1 | AGCCTTTC | 8 | - | - | + | - | - | + | - | - | + | - | - | - | - | - | + |
| 2/C6/11 | AGCCTTTC | 8 | - | - | + | - | - | + | - | - | + | - | - | - | - | - | + |
| 2/C5/2 | AGCTCTCC | 9 | - | - | + | + | - | - | - | + | - | + | - | - | - | - | + |
| 2/C5/17 | AGCTCTCC | 9 | - | - | + | + | - | - | - | + | - | + | - | - | - | - | + |
| 4/C5/3 | AGTCCTTC | 10 | + | - | - | + | - | - | - | + | - | - | - | - | - | - | + |
| 4/C5/4 | AGTCCTTC | 10 | + | - | - | + | - | - | - | + | - | - | - | - | - | - | + |
| 3/C5/8 | AGTCCTTT | 11 | - | - | + | - | + | - | - | + | - | - | - | - | - | - | + |
| 4/C5/1 | AGTCTTTT | 12 | - | - | + | - | + | - | - | - | + | - | - | - | - | - | + |
| 4/C5/2 | AGTCTTTT | 12 | - | - | + | - | + | - | - | - | + | - | - | - | - | - | + |
| 2/C5/16 | GACCCTCC | 13 | - | - | + | - | - | + | - | + | - | - | - | - | - | - | + |
| 3/C5/11 | GACCCTCC | 13 | - | - | + | - | + | - | - | - | + | - | - | - | - | - | + |

|  |  |  | ***E. faecium* antibiotic disc susceptibility profiles** | | | | | | | | | | | | | | |
| --- | --- | --- | --- | --- | --- | --- | --- | --- | --- | --- | --- | --- | --- | --- | --- | --- | --- |
|  |  |  | **tetracycline** | | | **ciprofloxacin** | | | **gentamicin** | | | **ampicillin** | | | **vancomycin** | | |
| **Sample No** | **SNP PROFILE** | **SNP ID** | **R** | **IR** | **S** | **R** | **IR** | **S** | **R** | **IR** | **S** | **R** | **IR** | **S** | **R** | **IR** | **S** |
| 3/C4/2 | GACCCTTT | 14 | + | - | - | - | - | + | - | - | + | - | - | - | - | - | + |
| 3/C4/5 | GACCCTTT | 14 | + | - | - | - | + | - | - | - | + | - | - | - | - | - | + |
| 3/C4/7 | GACCCTTT | 14 | + | - | - | - | - | + | - | - | + | - | - | - | - | - | + |
| 2/C6/6 | GATCCTTC | 15 | - | - | + | - | + | - | - | + | + | - | - | - | - | - | + |
| 2/C5/2 | GGCCCCCC | 16 | - | - | + | - | - | + | - | - | + | - | - | - | - | - | + |
| 3/C5/3 | GGCCCCCC | 16 | - | - | + | - | - | + | - | - | + | - | - | - | - | - | + |
| 3/C5/14 | GGCCCTCC | 17 | - | + | - | - | - | + | - | - | + | + | - | - | - | - | + |
| 3/C6/6 | GGCCCTCC | 17 | - | + | - | - | - | + | - | - | + | + | - | - | - | - | + |
| 3/C6/8 | GGCCCTCC | 17 | - | + | - | - | - | + | - | - | + | + | - | - | - | - | + |
| 3/C5/1 | GGCCCTTC | 18 | - | - | + | - | + | - | - | - | + | - | - | - | - | - | + |
| 3/C5/5 | GGCCCTTC | 18 | - | - | + | - | + | - | - | - | + | - | - | - | - | - | + |
| 2/C3/3 | GGCCTCCC | 19 | - | - | + | - | - | + | - | - | + | - | - | - | - | - | + |
| 2/C6/16 | GGTCCCCC | 20 | - | - | + | - | + | - | - | - | + | - | - | - | - | - | + |
| 2/C6/17 | GGTCCCCC | 20 | - | - | + | - | + | - | - | - | + | - | - | - | - | - | + |
| 2/C3/6 | GGTCCTCC | 21 | - | - | + | + | - | - | - | - | + | - | - | - | - | - | + |
| 2/C6/3 | GGTCCTCC | 21 | - | - | + | + | - | - | - | - | + | - | - | - | - | - | + |
| 2/C4/2 | GGTCCTTT | 22 | - | - | + | - | + | - | - | - | + | - | - | - | - | - | + |
| 2/C3/2 | GGTCCTTC | 23 | - | - | + | - | - | + | - | - | + | - | - | - | - | - | + |

^R^  Resistant , ^IR^  Intermediate resistant, ^S^ Susceptible
