# Supplementary material for: SNP diversity of Enterococcus faecalis and Enterococcus faecium in a South East Queensland waterway, Australia, and associated antibiotic resistance gene profiles
Source: BMC Microbiol. 2011 Sep 12;11:201. doi: 10.1186/1471-2180-11-201 (PMC3179957; doi:10.1186/1471-2180-11-201)
Supplement: Additional file 6 — Phenotypic and genotypic antibiotic resistance profiles of E. faecium isolated at each site. Antibiotic resistance profiles together with the E. faecium SNP profiles of strains isolated at all the sampling sites are listed here. [file 1471-2180-11-201-S6.DOC]

**Additional file 6 - Phenotypic and genotypic antibiotic resistance profiles of *E. faecium* isolated at each site**

| **Sampling sites** | **Sample No** | **SNP Profile** | **SNP ID** | **Antibiotic resistance profiles** |
| --- | --- | --- | --- | --- |
| **Santa Barbara(C3)** | 2/C3/3 | AAACTCTC | 1 | No antibiotic resistance detected |
|  | 2/C3/11 | AACCCTTC | 3 | No antibiotic resistance detected |
|  | 2/C3/4 | AACCCTTC | 3 | No antibiotic resistance detected |
|  | 2/C3/7 | AACCCTTC | 3 | Cip ^I.R^ [gyrA] |
|  | 2/C3/8 | AACCCTTC | 3 | Cip ^I.R^ [gyrA] |
|  | 2/C3/1 | AATCCTTC | 5 | No antibiotic resistance detected |
|  | 2/C3/5 | AATCCTTC | 5 | No antibiotic resistance detected |
|  | 4/C3/1 | AATCCTTC | 5 | Gen ^IR^ [aac(6')-aph(2')]; Cip ^I.R^ [gyrA] |
|  | 2/C3/3 | GGCCTCCC | 23 | No antibiotic resistance detected |
|  | 2/C3/6 | GGTCCTCC | 25 | Cip ^R^ [gyrA] |
|  | 2/C3/2 | GGTCCTTC | 27 | No antibiotic resistance detected |
| **Jabiru Island (C4)** | 2/C4/14 | AACCCTCC | 2 | No antibiotic resistance detected |
|  | 3/C4/6 | AACCCTTC | 3 | Amp ^R^ [pbp5]; Tet ^R^ [ tet M] |
|  | 2/C4/15 | AACTTTTC | 4 | No antibiotic resistance detected |
|  | 4/C4/1 | AGCCCTTC | 9 | Gen ^IR^ [aac(6')-aph(2')] |
|  | 3/C4/11 | AGCCTTCC | 11 | No antibiotic resistance detected |
|  | 3/C4/2 | GACCCTTT | 18 | Tet ^R^ [tet L,M & S] |
|  | 3/C4/5 | GACCCTTT | 18 | Cip ^I.R^ [gyrA]; Tet ^R^ [tet L & M] |
|  | 3/C4/7 | GACCCTTT | 18 | Tet ^R^ [tet L,M & S] |
|  | 2/C4/2 | GGTCCTTT | 26 | Cip ^I.R^ [gyrA] |
| **Paradise Point (C5)** | 1/C5/2 | AAACTCTC | 1 | No antibiotic resistance detected |
|  | 1/C5/4 | AAACTCTC | 1 | No antibiotic resistance detected |
|  | 3/C5/1 | AATCTTTC | 6 | No antibiotic resistance detected |
|  | 4/C5/5 | AGCCCCTC | 7 | Gen ^IR^ [aac(6')-aph(2')] |
|  | 4/C5/4 | AGCCCTTC | 9 | Gen ^IR^ [aac(6')-aph(2')] |
|  | 3/C5/10 | AGCCCTTT | 10 | No antibiotic resistance detected |
|  | 3/C5/6 | AGCCCTTT | 10 | Cip ^I.R^ [gyrA] |
|  | 3/C5/9 | AGCCCTTT | 10 | Cip ^I.R^ [gyrA] |
|  | 2/C5/1 | AGCCTTTC | 12 | No antibiotic resistance detected |
|  | 2/C5/17 | AGCTCTCC | 13 | Gen ^IR [^aac(6')-aph(2')];Cip ^.R^ [gyrA];Amp ^R^ [pbp5] |
|  | 2/C5/2 | AGCTCTCC | 13 | Gen ^IR [^aac(6')-aph(2')];Cip ^.R^ [gyrA];Amp ^R^ [pbp5] |
|  | 4/C5/3 | AGTCCTTC | 14 | Gen ^IR [^aac(6')-aph(2')]; Cip ^R^ [gyrA];Tet R [tet L & S] |
|  | 4/C5/3 | AGTCCTTC | 14 | Gen ^IR [^aac(6')-aph(2')]; Cip ^R^ [gyrA];Tet R [tet L & S] |
|  | 3/C5/8 | AGTCCTTT | 15 | Gen ^IR [^aac(6')-aph(2')]; Cip ^I.R^ [gyrA] |
|  | 4/C5/1 | AGTCTTTT | 16 | Cip ^I.R^ [gyrA] |
|  | 4/C5/2 | AGTCTTTT | 16 | Cip ^I.R^ [gyrA] |
|  | 2/C5/16 | GACCCTCC | 17 | Gen ^IR [^aac(6')-aph(2')] |
|  | 3/C5/11 | GACCCTCC | 17 | Cip ^I.R^ [gyrA] |
|  | 2/C5/2 | GGCCCCCC | 20 | Cip ^I.R^ [gyrA] |
|  | 3/C5/3 | GGCCCCCC | 20 | Cip ^I.R^ [gyrA] |
|  | 3/C5/14 | GGCCCTCC | 21 | Amp ^R^ [pbp5]; Tet ^R^ [tet(L),tet(M)&tet(S)] |
|  | 3/C5/1 | GGCCCTTC | 22 | Cip ^I.R^ [gyrA] |
|  | 3/C5/5 | GGCCCTTC | 22 | Cip ^I.R^ [gyrA] |
| **Coombabah (C6)** | 2/C6/10 | AACCCTTC | 3 | Cip ^I.R^ [gyrA] |
|  | 2/C6/15 | AGCCCTCT | 8 | No antibiotic resistance detected |
|  | 2/C6/13 | AGCCTTCC | 11 | No antibiotic resistance detected |
|  | 2/C6/11 | AGCCTTTC | 12 | No antibiotic resistance detected |
|  | 2/C6/6 | GATCCTTC | 19 | Cip ^I.R^ [gyrA]; Tet ^R^ [tet L & M] |
|  | 3/C6/6 | GGCCCTCC | 21 | Amp ^R^ [pbp5]; Tet ^R^ [tet(L),tet(M)&tet(S)] |
|  | 3/C6/8 | GGCCCTCC | 21 | Amp ^R^ [pbp5]; Tet ^R^ [tet(L),tet(M)&tet(S)] |
|  | 2/C6/16 | GGTCCCCC | 24 | Cip ^I.R^ [gyrA] |
|  | 2/C6/16 | GGTCCCCC | 24 | Cip ^I.R^ [gyrA] |
|  | 2/C6/3 | GGTCCTCC | 25 | Cip ^R^ [gyrA] |
